# Acceptability and feasibility of a community-based strength, balance, and Tai Chi rehabilitation program in improving physical function and balance of patients after total knee arthroplasty: study protocol for a pilot randomized controlled trial

**DOI:** 10.1186/s13063-021-05055-5

**Published:** 2021-02-11

**Authors:** Cathy W. T. Lo, Matthew A. Brodie, William W. N. Tsang, Chun-Hoi Yan, Priscillia L. Lam, Chun-Ming Chan, Stephen R. Lord, Arnold Y. L. Wong

**Affiliations:** 1 Department of Rehabilitation Sciences, The Hong Kong Polytechnic University, Hung Hom, Hong Kong SAR, China; 2 Neuroscience Research Australia, University of New South Wales, Randwick Sydney, Australia; 3 Department of Physiotherapy, The Open University of Hong Kong, Hong Kong SAR, China; 4 Department of Orthopaedics and Traumatology, The University of Hong Kong, Hong Kong SAR, China; 5 Department of Physiotherapy, Queen Mary Hospital, Hong Kong SAR, China; 6 Department of Orthopaedics and Traumatology, Yan Chai Hospital, Hong Kong SAR, China

**Keywords:** Total knee replacement, Falls, Tai Chi, Rehabilitation, Prevention, Multi-faceted intervention, Lower limb muscle strength, Balance, Coordination

## Abstract

**Background:**

The rate of falls in patients after total knee arthroplasty (TKA) is high and related to lower limb muscle weakness and poor balance control. However, since routine post-TKA rehabilitation is uncommon, it is paramount to explore alternative strategies to enhance balance and physical functioning in post-TKA patients. As Tai Chi is a proven strategy for improving balance in older people, the proposed study aims to determine the feasibility and acceptability of a 12-week community-based post-TKA multimodal Tai Chi program and to collect preliminary data with respect to the efficacy of such a program in improving balance and physical functioning in post-TKA patients as compared to usual postoperative care.

**Methods:**

A single-blinded 2-arm pilot randomized controlled trial will recruit 52 community-dwelling post-TKA patients (aged > 60 years) in Hong Kong. In addition, 26 untreated asymptomatic controls will be recruited for comparison purposes. The TKA patients will be randomized into either a 12-week multimodal Tai Chi rehabilitation group or a postoperative usual care group (26 each). Participants will perform the outcome assessments at baseline, 6, 12, 24, and 52 weeks after TKA, while asymptomatic controls will have the same assessments at baseline, 12, and 52 weeks after baseline. The rate of recruitment, retention, and attrition, as well as adherence to the intervention, will be measured and used to determine the feasibility of the study and whether a full-scale effectiveness trial is warranted. Further, qualitative interviews will be conducted to explore the acceptability and possible barriers to the implementation of the intervention. Primary and secondary outcomes including both patient-reported surveys and performance-based tests will be compared within and between groups.

**Discussion:**

The study will determine the feasibility and acceptability/potential efficacy of community-based rehabilitation for post-TKA patients and assess whether the intervention has the potential to be assessed in a future fully powered effectiveness trial. The findings will also be used to refine the study design and guide the conduction of a future definitive randomized controlled trial.

**Trial registration:**

ClinicalTrials.gov NCT03615638. Registered on 30 May 2018. https://clinicaltrials.gov/ct2/show/NCT03565380

**Supplementary Information:**

The online version contains supplementary material available at 10.1186/s13063-021-05055-5.

## Background

Total knee arthroplasty (TKA) is a well-established surgical intervention for patients with end-stage knee osteoarthritis (OA) to alleviate knee pain, correct knee deformity, and improve patients’ quality of life [1, 2]. However, research has consistently reported that the fall rate of post-TKA patients (up to 40%) is comparable to the pre-TKA fall rate (up to 50%) [3] and is higher than that of the age- and sex-matched asymptomatic controls [4, 5]. The persistent high fall rate may be partly ascribed to poor lower limb strength and the resection of soft tissues (e.g., the joint capsule) that contain numerous proprioceptors [6]. Such removal of proprioceptors may cause joint position sense deficits, increased muscle latency, and poor postural control [6]. Therefore, specific rehabilitation is needed to restore balance, movement control, and physical function of patients following TKA.

Recent evidence has shown that post-TKA, supervised progressive strength or balance training is better than inpatient care or home exercises in improving short- and long-term functional outcomes [6, 7]. Recent systematic reviews concluded that compared to standard postoperative care, supervised progressive postoperative lower limb strengthening exercises significantly improve knee flexion and extension range of motion, 6-min walk test (6MWT) distance, and timed up-and-go test (TUG) completion times [7, 8]. Similarly, post-TKA balance training (e.g., agility training) significantly improves balance and physical function of patients that reduces the risk of falls (as reflected from tests of stair climbing, functional reach, single leg stance, sit-to-stand ability, and the TUG) [7].

Unfortunately, routine postoperative outpatient physiotherapy or rehabilitation service is not always offered to patients after TKA, especially those in the sub-acute to chronic phases. Therefore, it is essential to explore alternative rehabilitation strategies to enhance lower limb muscular strength, improve balance, and reduce trip/fall incidences in patients following TKA. While conventional rehabilitation interventions comprising strengthening and dynamic exercises that have shown beneficial effects on pain and function after TKA [8, 9], community-based Tai Chi may provide an inexpensive, feasible, and effective option to enhance functional performance in post-TKA patients. Previous clinical trials have demonstrated beneficial effects of Tai Chi on lower limb muscle strength and balance in older people and stroke survivors in Hong Kong [10, 11]. One report has also shown that a 12-week Tai Chi program significantly improves pain, balance, physical functioning, quadriceps endurance, and femoral neck bone density in patients with knee OA as compared to untreated controls [12]. Additionally, a randomized controlled trial (RCT) involving 702 community-dwelling older adults in Australia [13] revealed that compared to untreated controls, 16 weeks of weekly community-based Tai Chi training significantly reduced falls (hazard ratio after 24 weeks = 0.67, 95% CI = 0.49–0.93) and improved five out of six balance measures. These findings highlight the efficacy of Tai Chi for fall prevention. Given the success of previous Tai Chi trials, it is plausible that community-based Tai Chi may improve balance, lower limb strength, and physical function in post-TKA patients.

Intervention programs comprising multiple components have been reported to be the most effective for improving a number of musculoskeletal and functional outcomes in older adults [14]. Numerous studies have evaluated individual components of post-TKA treatment and demonstrated the efficacy of the functional training including strengthening and balancing exercises [6, 9, 15], but there are few data on the effects of multimodal intervention programs that incorporate functional exercises with Tai Chi for post-TKA patients. The primary objective of this pilot study is to assess the feasibility of recruitment processes including the number of eligible participants and retention rates and to collect preliminary data to determine if a fully powered clinical trial is warranted.

The second objective is to determine the feasibility of a 12-week community-based post-TKA multimodal program and collect preliminary data with respect to the efficacy of the combination of different exercise components (i.e., including evidence-based strengthening and balancing exercises, and Tai Chi) in improving balance and physical functioning in post-TKA patients as compared to usual postoperative care. A third group of asymptomatic controls will be included to investigate whether the postoperative rehabilitation program can restore balance and mobility of TKA patients more akin to asymptomatic individuals. It is hypothesized that the multifaceted exercises is a feasible intervention for post-TKA patients, and those in the intervention group will demonstrate better physical function and balance control than patients receiving usual postoperative care.

## Methods

### Study design

**Fig. 1:**
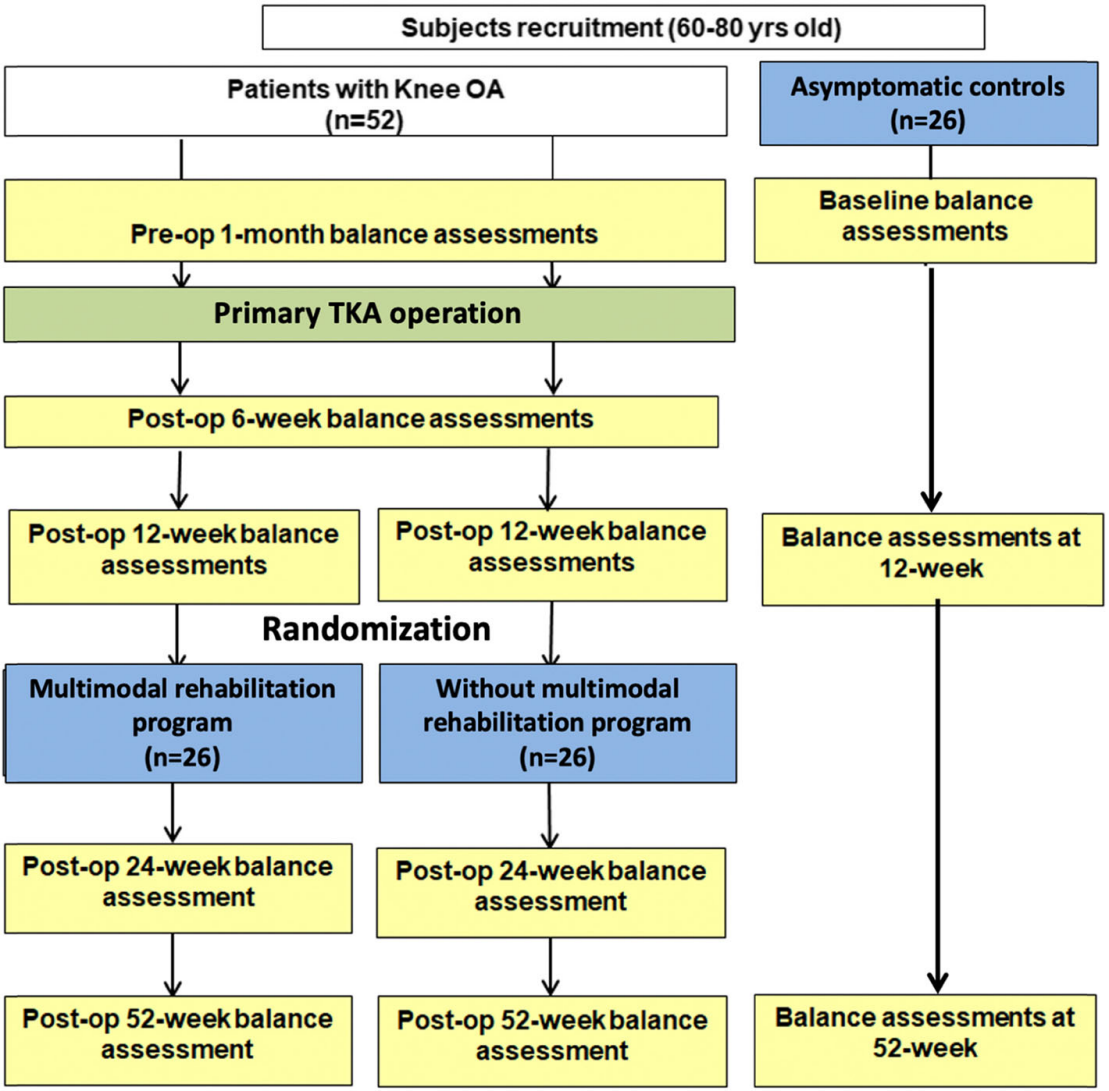
A flowchart of the single-blinded 2-arm randomized controlled trial and non-randomized asymptomatic control comparative study. The multimodal program will run once per week over 12 weeks

This is a single-blinded 2-arm RCT. TKA participants will be randomized to either a 12-week community-based rehabilitation program starting at 12 weeks after TKA, or usual postoperative care without outpatient physiotherapy. A third group of asymptomatic controls will be recruited to provide comparisons at various time points (Fig. 1). A third group of asymptomatic controls will be recruited to investigate whether the postoperative rehabilitation program can restore balance and mobility of TKA patients to the levels found in asymptomatic individuals (Fig. 1). All post-TKA participants will undergo five clinical assessments: 1 week before TKA, and at 6, 12, 24, and 52 weeks after TKA. Two orthopedic surgeons will use a standard parapatellar approach. Two common prosthesis designs (posterior stabilized and medial pivot) will be used in TKA surgery. Previous research has reported no significant differences in self-reported and physical performance outcomes between these prostheses 12 months after TKA [16]. In addition, the untreated asymptomatic controls will be assessed at baseline, and 12 and 52 weeks from baseline. This study protocol has been approved by the Human Research Ethics Committees at The Hong Kong Polytechnic University (HSEARS20171225001) and the Institutional Review Boards of two hospitals (UW 18-452) and has been prospectively registered on clinicaltrials.gov (identifier number: NCT03615638). The protocol has been developed in accordance with Good Clinical Practice, Standard Protocol Items: Recommendation for Interventional Trials (SPIRIT) (see Additional file 1 SPIRIT 2013 checklist). The Human Research Ethics Committees review all study activities annually including ethical conduct, regulatory compliance, and recruitment and retention and can initiate an independent study audit at any time. The investigator team meets biweekly to discuss enrolment targets and treatment fidelity. The study coordinator is responsible for ensuring that data is complete and monitoring study progress including sufficient recruitment and retention. Adverse events across conditions will be reviewed annually; issues with intervention delivery and other unintended consequences of either intervention are discussed during regular supervision meetings. Significant protocol modifications will be approved by relevant Institutional Review Boards and will be reported to relevant parties (e.g., ClinicalTrials.gov) in a timely manner. All participants have the option of discontinuing their participation in the study at any time, and this is indicated in the consent forms. Adverse events will be reported immediately to the principal investigator, tracked, and responded to according to regulatory guidelines. There are no criteria for the modification of the study interventions. As harm from this type of study is rare, there are no provisions in place for ancillary, post-trial, or compensation for study-related harms.

### Recruitment

Participants scheduled for TKA will be recruited from the osteoarthritis clinics of two hospitals in Hong Kong. It is anticipated that at least 10 TKA patients will be eligible for this study every month and the recruitment will take place over a 2-year period. A trained researcher will contact potential participants who are scheduled for TKA by phone to provide the required information regarding the current study (including objectives, eligibility criteria, and interventions). Those interested will be scheduled for an in-person visit to a physiotherapy department at one of the participating hospitals. A physiotherapist/trained research assistant will check the eligibility of the participant and assess cognitive function with the Mini-Mental State Examination [17], if this assessment has not been recently administered. After obtaining written consent, participants will undergo baseline assessments on the same day. Untreated asymptomatic controls will be recruited from community centers through posters and will undergo the same baseline assessments after obtaining informed consent by blinded physiotherapists/a trained research assistant at one of the two participating hospitals. The reasons for exclusion and for non-participation of eligible candidates will be documented in a screening log.

### Inclusion and exclusion criteria of participants

TKA participants will be eligible for this study if they are (1) aged 60 years or above, (2) planning to undergo primary TKA within 1 month, and (3) living independently in the community. People will be excluded if they are living in assisted living facilities, requiring nursing care, or planning to reside away from the hospital district within 1 year, or they have prior experience in practicing Tai Chi exercise for at least 3 months. Other exclusion criteria include the following: unstable medical conditions, fracture of lower limbs, malignancy in the last 5 years, lower limb prosthesis/amputation, congenital defect that is considered to cause the present complaint, systemic inflammatory and autoimmune diseases, previous osteotomy, neurological diseases (e.g., Parkinson’s disease, stroke), blindness, revision TKA, complications after primary TKA, and cognitive impairment as indicated by a Mini-Mental State Examination score < 19 [17]. Untreated asymptomatic controls will include a non-randomized cohort recruited using the same selection criteria with the exceptions that they must report no knee pain in the last 3 years, or have no history of TKA or total hip arthroplasty.

### Sample size

No formal sample size calculation was performed for this feasibility study. However, a minimum sample size for a pilot study to evaluate the feasibility of conducting a full-scale trial is suggested to be 50 per study [18, 19]. A total of 78 patients (26 per arm) were deemed an appropriate sample size for this trial to estimate the recruitment and retention rates and explore the acceptability of the intervention.

### Randomization and allocation

Upon completion of the 12-week post-operation assessments, TKA participants will be randomized into either the intervention or the usual care group. Permuted blocks using a computer-generated random number schedule will be used to determine the randomization order. Group assignment will be concealed in sealed sequentially numbered opaque envelopes and administered by a research assistant at The Hong Kong Polytechnic University not involved in the study. In addition, various methods will be employed to keep participant data confidential and secure including using password-protected files, limiting access to only those on the study team who require identifiable data, and using de-identified data when possible.

### Blinding

All assessments of TKA patients will be conducted by blinded physiotherapists/trained research personnel at one of the two participating hospitals. However, participants and Tai Chi instructor will not be blinded.

### Instructor

The researchers completed a Tai Chi certificate workshop for exercise leaders in Hong Kong provided by Dr. Paul Lam, who had originally developed the 12 movements of Sun-style Tai Chi exercise for arthritis. During the workshop, the intensity and forms of the 12 Tai Chi movements of exercise leaders were closely monitored to ensure that each movement would be applied correctly and safety to patients with arthritis. An illustrated training manual and reference guide along with the a standardized home-practice DVD (ISBN 978–0–9,803,573-1-8) was provided to the exercise leader prior to the study to guide them in performing each form of Tai Chi correctly.

### Interventions

#### Community-based multi-modal rehabilitation program group

Participants will receive 12 × 90 min sessions of supervised exercises over 12 weeks. In addition to usual care, supervised exercise will be held at the Center of Sports Training and Rehabilitation inside the University and each class will be restricted to no more than 8 participants to ensure sufficient personalized attention. Each training session involves warm-up together with stretching of lower extremity muscles (quadriceps femoris, hamstring, and calf muscle stretching) (10 min), strengthening exercises of the major lower extremity muscle groups by Theraband (isometric knee extensions in sitting/standing position, hip abduction, standing hamstring curls, and wall squat) (15 min), balancing exercise (side stepping, front crossover steps during forward/backward and laterally ambulation, back crossover steps during backward ambulation, shuttle walking, tandem walk, and standing on the foam surfaces) (15 min), Tai Chi training (20 min), and rest/ cool-down (15 min). All lower-extremity exercises will be performed bilaterally. The details of strengthening and balancing exercises are based on a previously published protocol [20], while the 12-form Sun style of Tai Chi is based on the “Tai Chi for Arthritis” program which involves six basic core movements and six advanced movements [12]. The 12 tai chi movements combine diaphragmatic breathing and relaxation with gentle and slow movements, both isometric and isotonic, while maintaining good postures. Tai chi exercise involves backward and forward movements with full-weight bearing on both lower extremities and qigong exercise with deep diaphragmatic breathing [12]. The written protocol with details of the intervention has been prepared and will be used by the exercise instructor.

Exercises are initially started at low intensity as indicated by the rate of perceived exertion (9–10 in the Borg scale 6–20) [21] and are progressively increased based on the overload principle [22], as long as participants do not experience significant increases in pain or discomfort (relative to the same day before the exercise class), increased effusion, or decreased range of knee motion following the exercises [23]. Intensity will be progressed based on individual ability, i.e., each participant will be monitored so that the difficulty of Tai Chi movements can be adjusted by varying the movement speed, magnitude of trunk rotation and weight shifting, base of support, and forward-backward movement with full weight-bearing on both lower extremities [12]. Each participant will be given both printed material and online videos about the Tai Chi exercises (including Tai Chi principles, practicing techniques, and safety precautions) (Additional file 2). Participants will be instructed to practice the same exercises at least 30 min daily to achieve 3 h of exercise per week [24]. The duration will be recorded in an exercise logbook, which will be checked every week to encourage adherence and to monitor the correct dosage. The logbook will be distributed to the participants at the beginning of the program and collected by the instructor at the last session. The Template for Intervention Description and Replication (TIDieR) Checklist has been adopted as a guideline for reporting the details of the intervention [25]. The fidelity of the intervention delivered by the instructor will be monitored and assessed by audio-recordings of sessions every 2 to 3 weeks. The principal researcher will listen to the voice clip and check whether the essential treatment components are included in each session using a checklist.

Upon completion of the supervised program, participants will be instructed to continue the exercises for 30 min per day at home until the postoperative 36-week follow-up (12-week home exercise). Previous research has recommended that fall prevention exercise should be no less than 50 h over 25 weeks to reduce fall risk in older people [24, 26]. The proposed exercise intensity will be 72 h over 24 weeks (12-week supervised exercise + 12-week home exercise). Further, the research personnel will contact participants by phone once every 2 weeks between the end of the 12-week program and the postoperative 36-week follow-up to facilitate their exercise adherence and document their weekly practice duration. Participants will be given supermarket coupons up to the value of HK$600 upon completion of all the 12-week intervention and 5 assessments.

#### Usual postoperative care group (Fig. 1)

Participants in the usual care arm will receive standardized usual inpatient postoperative care provided by the two participating hospitals and a total knee replacement pamphlet. The pamphlet contains information about early-stage postoperative exercises including lower limb stretching and mobilization exercises. In addition, some patients may be referred to outpatient physiotherapy services at the discretion of the physiotherapy or orthopedic team upon discharge or medical follow-ups. Upon the completion of all 5 assessments, participants will be given a maximum compensation of HK$250 supermarket coupons*.*

#### Untreated asymptomatic controls

To minimize the number of physical assessments for untreated asymptomatic controls, this group will undergo only three clinical assessments: at baseline and at 12 and 52 weeks after baseline (Fig. 1). Participants will be given a total possible compensation of $150 supermarket coupons only after the completion of 3 assessments.

## Endpoints and outcome measures

### Primary endpoint

The primary endpoint is to assess the feasibility and acceptability of trial design and procedures, including the recruitment and retention rates and identifying barriers to participation. This will inform the choice of the primary outcome for a full-scale trial and provide an estimation of the attrition rate for a sample-size calculation. The following will be measured and used to inform study feasibility:

#### Rate of recruitment and rate of retention

The recruitment rate will be calculated as the percentage of people who were screened for their eligibility for participating in the study and subsequently provided informed consent. The attrition rate will be calculated as the percentage of those recruited who did not complete reassessments at subsequent follow-ups. The recruitment and attrition rates will be monitored on a monthly basis to determine the presence of any specific trends. The recruitment rate (all potential participants), retention and attrition rates (all randomized participants), and completion rates (all enrolled participants) will be assessed at the end of study. Reasons for ineligibility and/or non-recruitment of eligible patients will be recorded and grouped into categories. Adverse events in all groups will be monitored, recorded, managed, and followed up. Participants will be given information regarding the procedure for reporting or handling of adverse events (e.g., a dedicated phone line for contacting the research team). All participants will have the right to withdraw from the study at any stage. The reasons for withdrawal will be documented and any data already collected from the participant will not be analyzed.

For participants who need to postpone their surgery after the baseline assessments, they will be reassessed 1 week before the rescheduled surgery. Participants who need to cancel their surgery after the baseline assessments will be treated as dropout cases and the corresponding reasons for non-participation will be documented. This procedure will enable the estimation of the recruitment rate and the sample size for the definitive trial.

#### Acceptability of the intervention and barriers to participation

It is important to explore barriers to participation during feasibility work because unforeseen challenges of recruitment can lead to early termination of definitive trials [27]. Attendance and exercise logbooks will be used to monitor non-adherence. The instructor will communicate with non-adherent participants to understand the reasons and barriers for non-adherence and to develop appropriate strategies to improve adherence (e.g., scheduling another time of day for performing home exercises, or reverting to the Tai Chi moves from the last week if the new move is too challenging, etc.). In addition to monitoring adherence, exercise class sessions will be randomly audio-recorded. The principal researcher will listen to the audio-recordings and document absence or presence of core parts of the intervention to check intervention fidelity.

Furthermore, to assess the acceptability of the intervention, each participant in the intervention group will be invited to participate in face-to-face or video call semi-structured interviews based on participants’ preferences within 1 month after the final follow-up. Visual materials such as video or photos of the exercise will be shown to the participants at the beginning of the interview to help them recall the content of the intervention program. The interviews aim to deepen our understanding of the barriers and facilitators to their participation in the intervention, and ways to improve adherence. All interviews will be audio-recorded after obtaining informed consent.

#### Sample size estimation for the future definitive trial

While the current feasibility trial is not powered to determine the efficacy of interventions, its findings will help estimate the standard deviation (SD) of the primary outcome (The Chinese version of Knee Injury and Osteoarthritis Outcome Scale (KOOS)) so as to inform the sample size calculation for the effectiveness trial. There are no plans to conduct interim analyses, i.e., while participant recruitment is on-going or before the final assessments are completed.

### Secondary endpoints

During the baseline assessments (1 week before TKA or baseline), participants will provide their demographic information and complete a set of questionnaires with the help of a research assistant if required (Table 1). The same set of questionnaires and assessment procedures will be repeated at the 6-, 12-, 24-, and 52-week post-TKA follow-ups in the TKA groups, and at the 12 and 52 weeks from baseline for the untreated asymptomatic controls.

**Table 1:** Outcome measures of secondary endpoints at different time points for participants

Types of measure	Assessments	Measurement time				
		Pre-op (Baseline)	Postop 6 wk	Postop 12 wk	Postop 24 wk	Postop 52 wk
**Subjective assessments**						
Sociodemographic characteristics	Age, sex, weight, height, dominant leg, marital status, number of people in the household, smoking habit, educational level, work status (number of working hours per week), occupation, alcohol/smoking status, history of trips/ falls, elevator landing or non-elevator landing flat	x				
Comorbidities	List of diseases: respiratory disease, hypertension, cardiovascular disease, eye problems, hallux valgus, previous hip or knee arthroplasty	x				
Pain	11-point numeric pain rating scale [28]	x	x	x	x	x
Physical function	Chinese version of Knee Injury and Osteoarthritis Outcome Score [29]	x	x	x	x	x
Depression	Chinese version geriatric depression scale [30]	x	x	x	x	x
Physical activity	Physical Activity Scale for the Elderly (Chinese version) [31]	x	x	x	x	x
Global impression of change in the intervention/ usual care	Self-perceived global changes				x	x
Fear of falling	Falls Efficacy Scale [32]	x	x	x	x	x
**Physical assessments**						
Range of motion	Lower limb joint range of motion by goniometer	x	x	x	x	x
Balance	Brief-Balance Evaluation System test [33]	x	x	x	x	x
Static and dynamic stability	Functional tests using two wearable inertial sensors during tandem stance, a timed up-and-go test, and a 6-min walk test.	x	x	x	x	x
Physical activity over 3 days	Activity monitoring for 3 days in a week	x		x	x	x

Note: The asymptomatic controls will only undergo follow-ups at 12-week and 52-week after the baseline

*TKA* total knee arthroplasty, *mth* month, *post-op* post-operation, *pre-op* pre-operation, *wk* = week

### Primary outcomes for a future definitive trial (Table 1)

#### Self-reported physical function

The Chinese version of Knee Injury and Osteoarthritis Outcome Scale (KOOS) will be used to document self-reported physical function and to evaluate knee status before/after TKA [29].

#### Number of trips/falls

The number of trips, falls, and fall-related injuries in the 12 months before baseline will be documented. During the follow-up, trips/ falls will be recorded using a trip/fall diary.

### Secondary outcomes for a future definitive trial (Table 1)

#### Objective measures of static and dynamic stability

Three small wireless and wearable inertial motion sensors (OPAL, APDM Inc., Portland, USA; sampling frequency of 128 Hz) will be used to assess the static and dynamic balance of participants during functional tasks. When wearing the sensors, participants will perform a tandem stance test with eyes open and eyes closed, the TUG and the 6MWT. This method has been found to be useful in characterizing gait stability of people with and without Parkinson’s disease [27, 34] and discriminating older fallers from non-fallers [35].

#### Knee pain

The NPRS is a gold standard for pain measurement [36, 37] given its high reliability, sensitivity and validity [28].

#### Depressive symptoms

Depressive symptoms will be examined by the short form Chinese version of the Geriatric Depression Scale (GDS), which comprises 15 questions [30].

#### Physical activity levels

Physical activity will be assessed by the Physical Activity Scale for the Elderly (Chinese version), which is a 12-item questionnaire that documents leisure, physical, household, and work-related activities over the last 7 days [31].

#### Fear of falling

Concern of falling will be measured with the Falls Efficacy Scale (FESI) [32], which showed good reliability and validity in measuring fear of falling in frail older people [38].

#### Global impression of change

Symptom severity, treatment response and the efficacy of treatment 24 weeks post-TKA will be measured by the Global Impression of change scale.

#### Joint range of motion

The ranges of motion of hip, knee, and ankle joints will be evaluated using a goniometer. These assessments are chosen because limited knee/ankle joint ranges have been reported as potential risk factors for falls [5].

#### Balance assessment

The Brief-Balance Evaluation Systems test comprises six static and six dynamic tasks of different difficulty levels [33].

#### Remote monitoring of physical activity

To quantify physical activity levels under free-living conditions, each TKA participant will wear an activity monitor to track physical activity over 72 h (from Thursday night to Sunday night) at 1 week before TKA, and again for another 3 days during the 12th, 24th, and 52nd weeks after TKA. Similarly, asymptomatic controls will undergo physical activity monitoring using the sensor for a 72-h period week following baseline, and during 12th and 52nd weeks of follow-up.

## Statistical analysis

The data regarding the feasibility, acceptability, adverse events, intervention adherence, missing data rates and data completion rates will be summarized using descriptive statistics. The experience and perception of participants toward the RCT solicited from semi-structured interviews will be analyzed by framework analysis [39, 40].

To determine the feasibility of studying the proposed outcomes in a definitive trial, inferential analysis will be conducted at 95% confidence intervals, and the significance level will be set at 0.05. The effect size of each intervention outcome will also be estimated. Descriptive statistics will be used to summarize patient demographic data. Baseline demographic and clinical variables (such as BMI, medication, number of comorbidities) in the three groups will be compared using analyses of variance (ANOVAs) and Kruskal-Wallis tests for parametric and nonparametric data, respectively. Any baseline variables that show significant between-group differences will be used as covariates in subsequent between-group analyses of outcome measures. Separate two-way repeated measures analyses of covariate (ANCOVA) or ANOVA will be used to evaluate within- and between-group changes in various outcome measures at several follow-up time points (i.e., 6, 12, 24, and 52 weeks after TKA for TKA patients, and 12 and 24 weeks after baseline for asymptomatic controls) in the three groups. Statistical analyses will be conducted using SPSS version 21.0 (IBM Corporation, NY, USA). Following the intention-to-treat principle, between-group differences in primary and secondary continuous outcomes in all randomized participants at different time points will be analyzed. Per-protocol analysis will also be reported for all planned outcomes in participants who strictly followed the protocol.

## Discussion

Total knee arthroplasty has been the most common surgery for treating patients with end-stage knee osteoarthritis since early 1980 [41]. Although multiple studies have shown that TKA improves patients’ pain and physical function [1, 2, 42], up to 60% of post-TKA patients experience falls within the first year after surgery [5, 43, 44]. Previous research has highlighted that patients at 1 year after TKA still display poor walking performance and balance compared with age- and sex-matched asymptomatic controls [16]. A recent systematic review has highlighted some modifiable risk factors (such as reduced knee range of motion and lower limb muscle weakness) that may increase the risk of falls in post-TKA patients [45]. While post-TKA comprehensive postoperative rehabilitation programs are recommended to restore balance and reverse other substantial physical deficits secondary to lower extremity muscle weakness and deconditioning [7, 45], current clinical practice includes little in the way of post-discharge interventions for TKA patients [46, 47]. Although supervised progressive strengthening or balance training can improve function and balance of patients with knee OA or TKA [6, 15], it may be difficult and costly to rehabilitate TKA patients in outpatient physiotherapy clinics for a prolonged period after discharge. While regular exercises can improve physical, psychological, and mental health of older people with and without TKA [30, 48], it may be difficult for patients with TKA to initiate regular exercises without proper guidance or community support. The high cost of outpatient rehabilitation services may deter older TKA patients from receiving postoperative rehabilitation. Community-based exercises such as Tai Chi may offer a feasible and affordable option for patients with TKA. Tai Chi can be learnt and practiced anywhere and anytime without the need for special clothing. More importantly, Tai Chi has demonstrated beneficial effects in reducing falls in patients with knee OA and stroke survivors [11, 49] and can improve lower limb muscle strength, balance, and physical function, which ultimately leads to improvements in balance and reduced risk of falling [12, 50]. To date, no studies have investigated the effects of community-based Tai Chi rehabilitation on balance and physical function in patients with TKA or intervention.

While interventions with multi-components have shown a beneficial effect on physical function in community dwelling older adults, none has evaluated a multifaceted approach incorporating a community-based exercise for post-TKA. This pilot randomized controlled trial will therefore evaluate the feasibility of a 12-week postoperative multimodal rehabilitation program (including evidence-based strengthening and balancing exercises, and Tai Chi) in improving balance and function of patients with TKA. We hypothesize that post-TKA patients in the intervention group will have superior physical function and balance control than post-TKA patients in the usual care group. Further, we hypothesize balance of the intervention group participants will approach the observed performance of asymptomatic controls. Since Tai Chi is widely accepted by Chinese in Hong Kong, this community-based Tai Chi rehabilitation strategy is likely to be well received by many post-TKA patients if it is proven effective in improving physical function and balance of TKA patients. The results of this study will assist in identifying the requirements of a community-based multifaceted rehabilitation for TKA patients as a definitive clinical trial, which may lead to a paradigm shift in rehabilitating post-TKA patients. In addition, the findings of this study will be disseminated to researchers and the public through the study’s entry on ClinicalTrials.gov, through publication in peer-reviewed journals, and through presentation of the findings to the scientific community at scientific conferences.

A cost-effectiveness analysis will not be conducted in the current study. However, incorporating health economic evaluations alongside RCTs are increasingly common to evaluate the cost-effectiveness of novel interventions. Healthcare costs related to the use of healthcare (e.g., number of doctor visits, number of hospitalizations, informal care such as assistance with personal care and household tasks), the cost related to the physical exercise program (costs of instructor and equipment), and quality of life should be identified and taken together to investigate whether the proposed intervention is a cost-effective treatment option*.*

## Trial status

This is the protocol version number 3 (8 August 2020). Recruitment began on 1 November 2018, and the recruitment will be completed on 1 November 2020.

## Supplementary Information

**Additional file 1.**

**Additional file 2.**

## Data Availability

Not applicable, no datasets are included in this study protocol.

## Abbreviations

6MWT: 6-min walk test
FESI: Falls Efficacy Scale
GDS: Geriatric Depression Scale
KOOS: Knee Injury and Osteoarthritis Outcome Scale
NPRS: Numeric pain rating scale
OA: Osteoarthritis
RCT: Randomized controlled trial
TUG: Timed up-and-go test
TKA: Total knee arthroplasty
