# Supplementary material for: Acceptability and feasibility of a community-based strength, balance, and Tai Chi rehabilitation program in improving physical function and balance of patients after total knee arthroplasty: study protocol for a pilot randomized controlled trial
Source: Trials. 2021 Feb 11;22:129. doi: 10.1186/s13063-021-05055-5 (PMC7877189; doi:10.1186/s13063-021-05055-5)
Supplement: Supplementary file 2 — Additional file 2. [file 13063_2021_5055_MOESM2_ESM.docx]

<https://connectpolyu-my.sharepoint.com/:w:/g/personal/17903518r_connect_polyu_hk/EYKz5B1FXjhLh0D_iNMt03wBaFDWW3axI2Yxp0gxbR1O2Q?e=SAsR8v>
